# Policy interventions to improve rural retention among neurosurgeons in Iran: A discrete choice experiment

**Published:** 2015-10-07

**Authors:** Sima Rafiei, Mohammad Arab, Arash Rashidian, Mahmood Mahmoudi, Vafa Rahimi-Movaghar

**Affiliations:** 1 Department of Health Management, School of Health, Qazvin University of Medical Sciences, Qazvin, Iran; 2 Department of Management and Health Economics, School of Public Health, Tehran University of Medical Sciences, Tehran, Iran; 3 Department of Epidemiology and Biostatistics, School of Public Health, Tehran University of Medical Sciences, Tehran, Iran; 4 Sina Trauma and Surgery Research Center, Sina Hospital, Tehran University of Medical Sciences, Tehran, Iran

**Keywords:** Discrete Choice Experiment, Stated Preference, Health Manpower, Rural Areas

## Abstract

**Background:** Health workforce shortages in rural and remote areas are a global challenge that almost every health system has to deal with. This study aimed to discover neurosurgeons’ job preferences and propose policy interventions that could possibly increase their retention in rural, remote, or underserved areas.

**Methods: **A discrete choice experiment (DCE) was conducted in November 2014 with a sample of Iranian neurosurgeons selected from five contrary’s provinces representing the geographical diversity. Job attributes included income, dual practice opportunities, workload, proximity to family, clinical infrastructure, housing, educational facilities, and work location. Probit regression model was used to estimate the importance of different job attributes and examine the extent to which neurosurgeons were willing to tradeoff between monetary and nonmonetary attributes.

**Results: **Findings indicated that increased salary, permission to undertake dual practice and access to adequate clinical infrastructure were the most important retention policies. Provision of subsidized housing and educational facilities also increased neurosurgeons’ attraction and retention in rural areas.

**Conclusion:** A range of policy interventions focusing on both monetary and nonmonetary incentives are required to increase neurosurgeons’ retention in rural, remote, or underserved areas.

## Introduction

Inequitable distribution of physicians between large metropolitan cities and remote or noncapital areas has become a serious concern and a priority to deal with.^1^^,^^2^ Despite being a universal concern,^3^^,^^4^ low and middle income countries made great efforts to give a best possible answer to the question of how to attract physicians and improve their retention in underserved areas.^5^^,^^6^ World Health Organization (WHO) has identified 16 retention strategies, including regulation and compulsory policies, financial motivations, education and training, personal and job-related support plans.^   7 ^ All around the world different policies have been developed to cope with uneven distribution of physicians. Some were related to monetary incentives^   8 ^ and some nonfinancial motivations,^   9 ^ although the successfulness of strategies were highly dependent on the local context and type of health cadres.^1^^,^^10^^,^^11^ 

One method to determine effective strategies for attracting and retaining health workforce in underserved areas is discrete choice experiment (DCE). This method has recently been used in health care as a form of stated preferences methods.^12^^,^^13^ DCE helps policy makers to determine a ranking of health providers’ preferences toward possible incentive packages in a way that each attribute’s value is comparable to another.^   14 ^ Recently, DCE has increasingly been used in several developing countries for rural retention problems.^5^^,^^15^^-^^19^ 

Islamic Republic of Iran is a middle income country located in south west of Asia. Iran Ministry of Health and Medical Education has recently faced with challenges in attracting and retaining health professionals particularly physicians in rural and remote areas. Nearly, 2000 general practitioners and 1700 specialists are lacking to serve in such areas of the country.^   20 ^ In addition, there are around 2200 specialists working in 270 underserved areas who are not satisfied with their income and work condition and seek an opportunity to leave their workplace in a shortest time possible.^   21 ^ Therefore, a particular attention must be paid to this issue and appropriate action plans should be proposed to encourage physicians to serve in rural, remote and nonmetropolitan areas of the country. To this aim, we conducted a DCE with technical assistance from WHO guidelines to inform the selection of appropriate recruitment and retention strategies based on physicians’ preferences.^22^^,^^23^ In this study, we chose neurosurgeons as they play important role in diagnosis, treatment and rehabilitation of neuro-spine illnesses or injuries and their shortage in some areas would cause irreparable damages.

The study objective was to examine physicians’ preferences for different job attributes and to find out important determinants for deciding to serve in a rural remote area. We also evaluated the effects of different policy interventions on the probability of choosing to serve in rural or underserved areas.

## Materials and Methods

A DCE was conducted in November 2014 with a sample of Iranian neurosurgeons selected from five contrary’s provinces representing the geographical diversity. Neurosurgeons under study were asked to choose between hypothetical job profiles describing as a combination of job attribute levels.

In the DCE literature, it is suggested to have at least 50 participants for each subgroup of interest.^17^^,^^24^^-^^26^ Following this rule, we considered two main subgroups of interest: those under 35 compared to those over 35, and those with rural background compared with those with urban background. Regarding to potential difficulties in data collection we increased target sample size by 20%, which led to 120 neurosurgeons. We used three stage sampling. First, five provinces were selected in a way to include geographical diversity. Second, remote and nonurban areas of each province where neurosurgeons were working in were defined and all included in the study. In the last stage, all in service neurosurgeons from public facilities were selected to reach the target sample.

Prior to conduct DCE, 17 in-depth interviews were done with in service neurosurgeons working in nine provinces of Iran to generate job attributes and corresponding levels from their own point of view. Physicians were asked about factors that could be influential in their retention in nonmetropolitan and remote areas.^   27 ^ The qualitative data were used to decide on attributes and corresponding levels. Levels of each attribute were created in a way to reflect the existing work condition. Then additional levels were produced from a rational increase in the base levels. Appendix 1 depicts job attributes and levels determined as a result of the qualitative study.

Once the eight attributes and related levels were determined, alternative job profiles were generated (25.9.4 = 900) consisting of all possible combinations of attribute levels. To reduce this to a practical number, SPSS software (version 22, SPSS Inc., Chicago, IL, USA) orthoplan procedure was used and produced a fractional factorial design with 16 scenarios.^24^^,^^28^ The criteria of level balance, orthogonality and minimum overlap among attribute levels were considered in designing an efficient experimental design.^   29 ^ One of the job profiles in an urban city with average attribute levels was selected as a constant scenario comparing to the remaining job profiles and provided 15 choice sets for each physician to regard. The questionnaire also included a number of socio demographic questions to collect background characteristics of the physicians. The sequence of presenting job pairs and the attributes was different in the questionnaire to reduce any risk of careless response and boredom of respondents.^   30 ^ Face and content validity of the questionnaire was tested in a pilot study conducted on 17 neurosurgeons who had participated in the interviews to identify any modification needed to be applied in a prior questionnaire. Final DCE questionnaire was applied to 120 in service neurosurgeons working in public health facilities of selected districts during November and December 2014. An example of a choice set is shown in table 1.

Ethical issues related to the research were taken in to account. Permission to conduct the research was obtained from Tehran University of Medical Sciences, Iran, and participation in the study was considered to be voluntarily.

Data were entered in SPSS and checked for consistency. To analyze survey results, questionnaire data were transformed in to STATA (version 13, Stata Corp LP, College Station, USA) wherein dependent variable (whether to choose Job A or Job B) was defined in a binary form. Probit regression model was used to analyze preference data and determine whether physician selected Job A or Job B. To investigate the correlation between physicians’ responses to different job choices, we used a random effect probit model. Estimated coefficients in the model equation would illustrate the marginal utility of each attribute level from physicians’ point of view. They also provided information about the significance and direction of the effects related to change in levels of attributes:

Prob [U job B > U job A] = β_1_ location + β_2_ income (2-4) + β_3_ income (4-5) + β_4_ income (5-6) + β_5_ dual + β_6_ workload (average-light) + β_7_ workload (average-heavy) + β_8_ family + β_9_ education + β_10_ clin + β_11_ housing (basic-none) + β_12_ housing (basic-superior) + ᶓ + µ

Where location, income (2-4), income (4-5), income (5-6), dual, workload (average-light), workload (average-heavy), family, education, clin, housing (basic-none) and housing (basic-superior) represent the difference in attribute levels between job A and B and corr (ᶓ , µ ) stands for the correlation among individual choices.

As evaluation of different policies to identify most effective interventions in physicians’ retention is one of the most interesting analyses for policy makers we used regression results to calculate willingness to pay (WTP) and policy impact measures in our study. First we measured the amount of monetary attributes physicians were willing to overlook in obtaining more of nonmonetary attributes using marginal rate of substitution (MRS). This way, WTP for each attribute was calculated by dividing the attribute regression coefficient by income coefficient. Although WTP could be calculated for all income levels, but we only reported it for the lowest level to best reflect current situation.

${\mathrm{MRS}}_{\mathrm{iq}}^{\mathrm{kh}}=-\beta_{h}|\beta_{k}$

Where ${\mathrm{MRS}}_{\mathrm{iq}}^{\mathrm{kh}}$ is the individual q’s marginal WTP for attribute h, β_h_ represents the coefficient of attribute h and β_k_ corresponds to the coefficient of attribute k (income) of the model.

Finally, we simulated the impact of various policy interventions on probability to choose rural job positions among physicians.^   30 ^ To do so, we considered a baseline job (a job with the lowest level of attributes) and estimated the change in the probability of taking it up following a change in one of the job attributes. Marginal effect analysis was conducted for the purpose.^   31 ^

## Results

In total, 120 neurosurgeons working in public hospitals of 5 country provinces’ 15 districts completed the questionnaire (response rate = 83.6%). Respondents were mainly male and married (96.7%), and had urban background (55.8%). All respondents have chosen the best job profile with superior attribute levels verifying the internal consistency of responses.

The results for random effects probit model on which job neurosurgeons would choose confirmed that regression coefficients were statistically significant for seven of the attributes (location, income, dual practice, workload, educational facilities, clinical infrastructure, and housing) verifying their importance for neurosurgeons to make decision about different job choices. We tested the theoretical validity of the model by checking out the attribute levels’ sign and determined whether they were in compatible with anticipated sign or not. At 5% of confidence interval level, physicians positively valued working in urban area, higher salary, opportunity for dual practice and more number of surgeries per month, having access to adequate clinical infrastructure, educational facilities, and proper subsidized housing. Relative impacts of the attributes were also estimated using partial log-likelihood analysis. 

**Table 1:** An example of a choice set

**Job A**	**Job B**
Location: Rural, remote or underserved area	Location: National capital or urban developed city
Income: 200% increase in current income	Income: 150% increase in current income
Dual practice: Yes	Dual practice: No
Workload: Heavy	Workload: Heavy
Proximity to family: No	Proximity to family: No
Clinical infrastructure: Adequate	Clinical infrastructure: Adequate
Educational facilities: Superior	Educational facilities: Basic
Housing: Basic	Housing: No

Which job profile do you prefer to choose? Job AO job BO

**Table 2:** Probit regression results and monetary value of different job attributes

**Independent variables**	**From … to …**	**Coef.**	**SD**	**P**	**WTP (%)**
Location	Urban to rural	-0.17	0.08	0.040	-8.5
Income 1	2000-4000 $	1.00	0.17	< 0.001	-
Income 2	4000-5000 $	1.29	0.15		-
Income 3	5000-6000 $	1.49	0.21		-
Dual practice	Not permitted to permitted	2.79	0.11		139.5
Workload 1	Low to moderate	0.37	0.08		18.5
Workload 2	Moderate to high	0.17	0.06	0.006	8.5
Family proximity	Near to far	0.10	0.08	0.200	-
Educational facilities	Basic to superior	0.20	0.08	0.001	14.0
Clinical infrastructure	Inadequate to adequate	0.70	0.11	< 0.001	36.5
Housing 1	None to basic	0.63	0.10		31.5
Housing 2	Basic to superior	0.58	0.11		29.0

WTP: Willingness to pay; SD: Standard deviation

**Table 3:** Estimated take up rates for a rural job under different policy options

**Independent variables**	**From … to …**	**Marginal effect**	**Take up rates (%)**	**P**
Location	Urban to rural	-0.046	4	0.040
Income 1	2000-4000 $	0.205	20	< 0.001
Income 2	4000-5000 $	0.280	28	
Income 3	5000-6000 $	0.335	33	
Dual practice	Not permitted to permitted	0.654	65	
Workload 1	Low to moderate	0.103	10	
Workload 2	Moderate to high	0.047	4	0.005
Family proximity	Near to far	0.028	-	0.200
Educational facilities	Basic to superior	0.080	8	0.001
Clinical infrastructure	Inadequate to adequate	0.178	18	< 0.001
Housing 1	None to basic	0.163	16	
Housing 2	Basic to superior	0.149	15	

Results showed that the most important attribute was dual practice, which physicians put the greatest value on it and the least important attribute was workload from their point of view.

The magnitude and sign of estimated attribute levels’ coefficients suggested that physicians regarded a higher level of utility for superior attribute levels except for workload that respondents preferred a moderate level rather than superior. The negative sign of location coefficient confirmed that respondents desired a job in urban area rather than rural, remote, or underserved town (Table 2). The sixth column of the table 2 reports the WTP estimates for different attribute levels. As data shown, neurosurgeons required a remuneration of 170 $ per month (8.5% of their income) to work in a rural area. They were willing to give up (2790 $ or 139.5% of income) to obtain the possibility of working in a private sector. To attain a moderate level of workload, having access to superior educational facilities and adequate clinical infrastructure, they were willing to sacrifice (370 $ or 18.5%), (280 $ or 14%) and (730 $ or 36.5%), respectively. In terms of housing, there was a WTP (630 $ or 31.5% of income) to be provided with basic subsidized housing.

The impact of improvement in the level of nonmonetary attributes as policy intervention on the probability to choose a rural job was also analyzed in the study. The marginal effect estimates in table 3 showed that an increase in workload from low to moderate level was associated with 10% increase in probability of choosing such a job.

Providing a chance to undertake dual practice and having access to basic subsidized housing would respectively raise the probability by 65% and 16%. If physicians were supposed to have access to superior educational facilities and adequate clinical infrastructure, 8 and 18% increase could be achieved in the likelihood of choosing a remote job. Raising the monthly income up to 4 and 5 thousand dollars increased the probability of taking a rural job by 20% and 28%. Finally, enlarging the salary up to 6000 $ enhanced the probability by 33%. The results showed that although wage increases were important incentives, but they became less effective as income increased. In addition to check for the effects of promotion in single attribute levels; we also examined the impact of different scenarios combined of a group of attribute levels on the probability of choosing a rural job among neurosurgeons. As table 4 depicts, a combination of 150% salary increase, opportunity for dual practice and access to adequate clinical infrastructure would result in 99% increase of rural uptake.

By providing the opportunity to access adequate clinical infrastructure and subsidized housing, rural uptake would increase up to 54%. If neurosurgeons in rural areas were provided with superior educational facilities and moderate level of workload, the uptake rate would be very similar to the effect of 200% increase in salary and providing a superior housing for physicians.

## Discussion

This study revealed important findings about neurosurgeons’ job preferences and policy interventions that could possibly influence their attraction and retention in rural, remote and underserved areas. Data analysis found that all job attributes except proximity to family had statistically significant effect on neurosurgeons’ preferences in their job choices. This finding implied that there could be a range of policy interventions to improve the probability of choosing a rural job among physicians. To consider an attractive job profile, physicians had strong preference to have opportunity for dual practice and to gain higher levels of salary. Besides monetary bonuses, there were some nonmonetary incentives, which played an important role in physicians’ recruitment and retention in underserved areas. Literature supports the findings and emphasizes on the importance of different nonmonetary factors such as opportunity to undertake dual practice, access to subsidized housing, adequate clinical infrastructure, and decent educational facilities as important issues in physicians’ preferences.^5^^,^^15^^,^^32^^-^^37^ Rockers et al.^   33 ^ indicated that regulations in favor of dual practice would make rural jobs attractive. Another study acknowledged that lack of resources and medical equipment acted as a barrier for health workforce to accept a rural job.^   38 ^ Kolstad^16^ and Mangham and Hanson.^17^ considered decent housing as a main incentive to work in rural areas. Hanson and Jack^39^ recognized that physicians put the maximum value on the ability to work in private sector. Improved housing, adequate medical equipment, and reduced time commitment were other key factors in their decision to retain.

In our study, participants were willing to tradeoff between different job attributes. They were ready to give up some amount of income in return to obtain subsidized housing and opportunity for dual practice. To work in a national capital or large developed cities rather than rural or remote areas, they requested an increase in current level of income. Similar to our findings, Kolstad^16^ found that respondents were willing to give up some amount of income to work in a place with sufficient clinical infrastructure and subsidized housing. Mangham and Hanson.^17^ also confirmed that nurses would sacrifice some pay increases to obtain the opportunity for continuous education and basic government housing. 

The study findings revealed that individual incentives or mixtures of them could improve neurosurgeons’ recruitment and retention in rural or remote areas. Miranda et al.^40^ confirmed that combinations of incentives could lead to higher rates of attraction or retention in rural jobs. In a study conducted in Uganda with the purpose of determining retention policies to attract and retain health workforce in rural areas, Rockers et al.^33^ found that salary increase, quality improvement of health facilities and monetary support for continued education would make rural jobs more attractive. Kruk et al.^18^ in a study of rural practice preferences among medical students in Ghana declared that the joint of three non-financial attributes, including better housing, adequate infrastructure and shorter contract duration would increase the uptake rate of rural areas. A similar research in Zambia suggested government housing, adequate clinical infrastructure, car loans, and educational facilities as important role players for the purpose.^   41 ^

**Table 4:** Prediction of the uptake rate for rural jobs under different policy scenarios

**Policy scenarios**	**Take up ** **rates (%)**	**P **
Permission for dual practice + 150% salary increase + adequate clinical infrastructure	99.0	< 0.001
Permission for dual practice + subsidized/government housing	96.0	
Adequate clinical infrastructure + subsidized/government housing + Permission for dual practice	98.0	
100% salary increase + permission for dual practice + subsidized/government housing	97.0	
100% salary increase + moderate workload + subsidized/government housing	52.0	
Adequate clinical infrastructure + subsidized/government housing	54.0	
Rural location + superior educational facilities + moderate workload	58.6	
200% salary increase + superior housing	58.4	

Vujicic et al.^14^ directed a research to estimate the impact of different policies on improving rural recruitment and retention. They recognized that increasing the amount of salary and possibility for long-term education could play an important role in increasing the take up rate of rural jobs.

***Strengths and limitations of the study***

There are some strength points regarding to this study. The fact that little studies have been conducted on health human resource management in developing countries,^   16 ^ we have attempted to close the research gap and provide valuable information about neurosurgeons’ job preferences to work in rural areas. Second, we have assigned the attributes and related levels on the basis of information obtained from a qualitative study conducted among neurosurgeons. Third, we piloted the study with 17 neurosurgeons to ensure that the hypothetical alternatives were well defined and respondents understood how to make their choices. Finally, we collected information from those neurosurgeons currently in service to provide useful information about their perceived incentives rather than medical students or those who have not entered in to the work market yet.

On the other hand, some limitations could be mentioned for the study. First of all we limited our research to one group of specialists because of practical matters. Second, we did not consider costs information and monetary valuations of different policies to determine the most cost effective one affecting physicians’ job choices. Finally, because of Inaccessibility to experimental design software solutions to construct efficient designs for choice experiments, we used a constant comparator.

## Conclusion

The study findings recommended that in order to resolve geographical imbalances, policy makers should respect not only appropriate reimbursement, but also improvement in a number of non-financial job attributes in rural or remote areas. Analysis revealed some retention strategies to make rural jobs more appealing for neurosurgeons. Increase in salary, legislation in agree with dual practice, improvement in clinical infrastructure, providing subsidized housing and opportunity for continuous education were some of the main inputs suggested to be included in retention policy packages. As there are relatively inadequate numbers of researches on job preferences of health workforce in developing countries, this study can have a significant contribution to the literature.

**Appendix 1:** Job Attributes and levels

**Attribute**	**Definition**	**Level**
Location	This attribute provides an alternative for respondents to choose between two job profiles (rural and urban areas). Rural areas represent remote, underserved, and nonmetropolitan districts of the country. Urban areas stand for national capital, regional, or district headquarters	Rural Urban
Income	This is the income obtained from governmental sources such as salary, allowances, fee for service, but not those from private practice. Four levels had been defined for income attribute. First level represents the base income; the second, third, and the forth levels stand for 100%, 150%, and 200% increase in base level of income, respectively	Base income Base + 100% Base + 150% Base + 200%
Dual practice	This means whether physicians are permitted to work in private sector besides public facilities or not	No Yes
Workload	This attribute identifies three levels. Low level relates to 5-15 surgical operation per month, moderate level relates to 15-25 operation and high level relates to more than 250 operations per month	Light Moderate Heavy
Proximity to family	This attribute identifies whether physician has to work in a place apart from family or live together in a same place	No Yes
Educational facilities	This attribute is defined in two levels. Basic refers to having access to a general medical library with few specialized books and journals and wireless internet access. Superior level refers to availability of a specialized library with up to date scientific references and fast internet access also possibility to hold journal clubs and training sessions	Basic Superior
Clinical infrastructure	This attribute is defined in two levels. Basic refers to availability of simple diagnostic and treatment facilities in an area. Superior refers to availability of MRI, CT scan and specialized operating rooms	Inadequate Adequate
Housing	This attribute identifies three levels. None depicts a situation which government does not provide a free housing for physician. Basic refers to a situation which government provides a suite with shared kitchen and bathroom for physician. Superior refers to a situation which government provides an apartment with bedroom, kitchen, and bathroom	None Basic Superior

CT: Computed tomography, MRI: Magnetic resonance imaging

## Notes:

**How to cite this article: **Rafiei S, Arab M, Rashidian A, Mahmoudi M, Rahimi-Movaghar V. Policy interventions to improve rural retention among neurosurgeons in Iran: A discrete choice experiment. Iran J Neurol 2015; 14(4): 211-8.
